# Immersive Technologies in Forensic Mental Health and Prison Settings: Scoping Review

**DOI:** 10.2196/95581

**Published:** 2026-07-17

**Authors:** Ivana Nakarada-Kordic, Dilshani Kumarapeli, Lana Chisholm, Emma Marie Buitenhek, Stephen Reay

**Affiliations:** 1 Auckland University of Technology Auckland, Auckland New Zealand

**Keywords:** immersive technologies, virtual reality, forensic mental health, prisons, scoping review, participatory implementation

## Abstract

**Background:**

The application of immersive technologies, particularly virtual reality, has expanded rapidly across health care domains, including mental health, rehabilitation, and education. These technologies enable the creation of controlled, interactive, and ecologically valid environments that can support therapeutic interventions, skill development, and behavioral assessment. Within forensic mental health services (FMHS) and prison settings, where individuals often present with complex psychological needs in restrictive and highly regulated environments, immersive technologies offer potential advantages such as safe simulation of real-world scenarios, enhanced engagement, and personalized intervention delivery. However, despite increasing interest, the evidence base remains fragmented, and questions persist regarding effectiveness, ethical implications, and feasibility of implementation in secure and resource-constrained contexts.

**Objective:**

Interest in immersive technologies in FMHS and prison settings is growing, yet their role remains unclear. This scoping review mapped current uses, highlighted opportunities, and identified key gaps and considerations for future implementation.

**Methods:**

A scoping review of English-language publications (2010-2025) was conducted using the Scopus, PubMed, and CINAHL databases. Data extraction followed the Joanna Briggs Institute framework, and thematic analysis explored benefits, drawbacks, and implementation barriers.

**Results:**

Thirty sources were identified. Primary research focused mainly on virtual reality for therapy, skill training, education, and assessment. There was evidence suggesting benefits such as increased engagement, emotional regulation, skill acquisition, autonomy, and improved clinician-patient dialogue. However, the studies were small, heterogeneous, and inconsistently reported, with limited long-term follow-up. Implementation barriers included institutional, ethical, and technical constraints and limited personalization and end user involvement. Co-design and participatory approaches surfaced as key enablers of acceptability, relevance, and safe use.

**Conclusions:**

The existing evidence base is preliminary and exploratory but indicates that immersive technologies may have potential value in FMHS and prison contexts. Current findings should be interpreted cautiously because studies are small, heterogeneous, and rarely include long-term follow-up. More robust evidence, careful implementation, and meaningful end user input are needed to support safe, relevant, ethical, and effective use. The emphasis on coproduction and guidance for safe, user-centered implementation is a novel contribution.

## Introduction

Digital tools are redefining how health care is delivered and experienced [1]. Immersive technologies such as virtual reality (VR) and augmented reality hold the potential to promote health and well-being, but their adoption often depends more on context and user factors than on technological limitations [2]. These technologies create simulated experiences that users perceive as real through the provision of various sensory information [3]. Despite their potential, evidence is limited on how immersive technologies can be applied effectively in mental health care and even less so within the care-control continuum of forensic mental health services (FMHS) [4].

FMHS operate at the intersection of health care, criminal justice, and legal ethics [5]. FMHS provide assessment and treatment for people with mental illness who have a history of or are at risk of violent offending [6], aiming to reduce symptoms and reoffending, improve quality of life, and support reintegration where possible [7]. These services balance care and control, with compulsory treatment and restricted freedoms designed to protect both patients and others [8]. Service users often experience prolonged, intensive, and restrictive service engagement, making them among the most disadvantaged populations in mental health care.

FMHS are high-cost, high-risk, and low-volume settings yet must achieve high-value rehabilitative outcomes. They are criticized for inconsistent organization and variable evidence-based practice [9] and face mounting pressures from rising mental disorder prevalence [10], increasing demand for interventions, and global shortages of mental health practitioners [11]. These challenges highlight the need for innovative approaches that strengthen the quality, consistency, and accessibility of care.

Immersive technologies show promise for therapy and service delivery [4,12]. In FMHS settings, where service users often present with complex psychiatric needs and histories of transgressive behavior, ethical considerations are paramount. Barriers such as limited familiarity with technology, resistance to change, and apprehension about its use can hinder adoption [13]. Careful, phased introduction is essential to ensure safety, comfort, and therapeutic value [13,14], yet few protocols exist to guide the use of immersive technologies in these contexts [4].

This scoping review provides an overview of existing literature where VR and other forms of immersive technology were used, developed, or considered for use in the FMHS and prison contexts. Although different in their primary framework (ie, punishment vs treatment), we chose to add to our scope literature related to the prison setting due to its similarity to FMHS—both being secure institutions housing vulnerable and high-risk populations, with overlapping goals of safety, treatment and rehabilitation, and reintegration.

Existing reviews have focused on VR in health care broadly [15], psychiatric assessment and treatment [16], or education and training in prisons [17]. Other work has explored clinicians’ perspectives on immersive technologies in mental health care in general [12] or eHealth tools for therapeutic purposes in forensic contexts [18]. However, none have examined immersive technologies beyond VR or addressed implementation implications for vulnerable users in complex FMHS and prison environments or uses extending beyond clinical assessment and treatment in these contexts.

This review, therefore, addressed two questions:

1. What aspects has primary research involving immersive technologies in FMHS and prison settings sought to assess?
2. What are the benefits, barriers, opportunities, and unintended consequences of implementing immersive technologies in these settings?

## Methods

### Overview

A scoping review methodology was selected for this research as it is well suited to exploring emerging areas and identifying gaps in existing knowledge [19]. The research questions (RQs) reflect the Joanna Briggs Institute (JBI) aims for scoping reviews [20]: first, to map the evidence available within a given field (RQ 1) and, second, to examine the breadth of existing work to reveal gaps in the research base (RQ 2). Although systematic in its process, this approach uses descriptive and qualitative synthesis to provide a rapid overview of key concepts, current research approaches, and the types of evidence present in the literature [21]. No review protocol was prospectively registered because this was designed as an exploratory scoping review to map an emerging and heterogeneous evidence base rather than to test a narrowly specified intervention effect.

### Search Strategy and Data Sources

This review was based on English-language publications published from January 2010 to November 2025 in the Scopus, PubMed, and CINAHL databases using Boolean operators to create our search strings. These were constructed using the following keywords and their combinations: (“forensic mental health” OR “forensic psych*” OR prison*) AND (“immersive technolog*” OR “virtual reality” OR “extended reality” OR “augmented reality” or “mixed reality”) NOT adolescen* NOT teen* NOT youth NOT highschool* NOT high school* NOT “prisoner’s dilemma”).

In addition, the reference lists of key articles were searched. All results were imported to Covidence (Veritas Health Innovation) for screening, and duplicates were removed.

### Eligibility Criteria

Eligibility criteria were developed in line with the review questions and the JBI approach to scoping reviews. Sources were eligible if they focused on the use, development, or consideration of immersive technologies in adult forensic mental health or prison settings. This included VR and related technologies used for therapeutic, rehabilitative, educational, training, or assessment purposes. Because initial searches identified few primary studies in forensic mental health settings, the scope was expanded to include prison-based literature where immersive technologies were used in related rehabilitation, re-entry, or educational contexts. Consistent with the aims of a scoping review, a broad range of source types was included—primary studies, expert commentaries, and conference proceedings—to ensure comprehensive coverage where peer-reviewed empirical literature remains limited. Only English-language articles published between 2010 and November 2025 were included. Publications before 2010 were excluded due to the rapid development of immersive technologies. Study protocols were excluded in cases in which a corresponding full study was available. Because this review included both empirical and nonempirical sources, source type was recorded during data extraction and considered during synthesis. Empirical studies were used to map applications, outcomes, acceptability, feasibility, implementation experiences, and reported ethical or practical concerns. Commentaries and conceptual papers were used to supplement this evidence by helping contextualize broader ethical, legal, and human rights implications.

### Study Selection

Titles and abstracts were initially screened by EMB and LC in Covidence against the inclusion and exclusion criteria. After exclusion at this stage, the remaining articles were then assessed in full text by EMB and LC. Where there was uncertainty or disagreement at either stage, this was discussed with IN-K, SR, and DK to reach consensus. Duplicate records were removed, with peer-reviewed versions prioritized where relevant. Interrater reliability statistics were not calculated because the screening process was designed as a consensus-based scoping review process rather than as an agreement estimation exercise. To support consistency, reviewers applied predefined eligibility criteria, used Covidence to manage screening decisions, and discussed uncertain or discrepant cases with the wider review team until consensus was reached. The absence of formal agreement statistics is acknowledged as a methodological limitation as it limits the extent to which reviewer consistency during study selection can be independently assessed.

### Data Extraction Strategy

Data extraction was guided by the JBI *Manual for Evidence Synthesis* [20]. A structured data extraction form was developed in Microsoft Excel to record the main details of each included source (Multimedia Appendix 1 [13,14,22-49]). This included author and year, country, setting, type of immersive technology, degree of immersion, application or intervention, study purpose, participant characteristics, study design, outcome measures, and key findings. Where relevant and explicitly reported, notes were also made on implementation issues, usability, barriers and facilitators, end user involvement, personalization, and ethical considerations. Data extraction was completed by EMB and LC, and the spreadsheet was shared with the wider research team for review and discussion. Given the breadth of the review and the heterogeneity of the included literature, formal quality appraisal was not undertaken in keeping with the aims of a scoping review.

### Descriptive Synthesis

The primary research studies were summarized based on descriptive features, including the technology used, study focus, measured outcomes, and key findings. This provided an overview of the types of tools, applications, approaches, and outcomes currently studied in FMHS and prison settings.

### Thematic Synthesis

To explore the reported benefits, barriers, drawbacks, opportunities, and unintended consequences associated with immersive technologies in FMHS and prisons, a thematic approach was used to synthesize the extracted findings from the included sources. This was used alongside the descriptive synthesis to identify common patterns across the literature. EMB and LC first read and reread the extracted material, including study findings, author interpretations, and observations related to implementation, and developed the initial codes. These codes were then discussed with IN-K and SR and grouped into broader themes. The themes were further reviewed and refined by the wider review team to ensure that they reflected the range of material included in the review. The final themes were then defined and used to produce a narrative account of the main issues identified across the literature. Given the heterogeneity of the included sources, this process was intended to identify patterns in the literature rather than provide a detailed qualitative analysis of primary data.

## Results

### Overview of Primary Research

**Figure 1:**
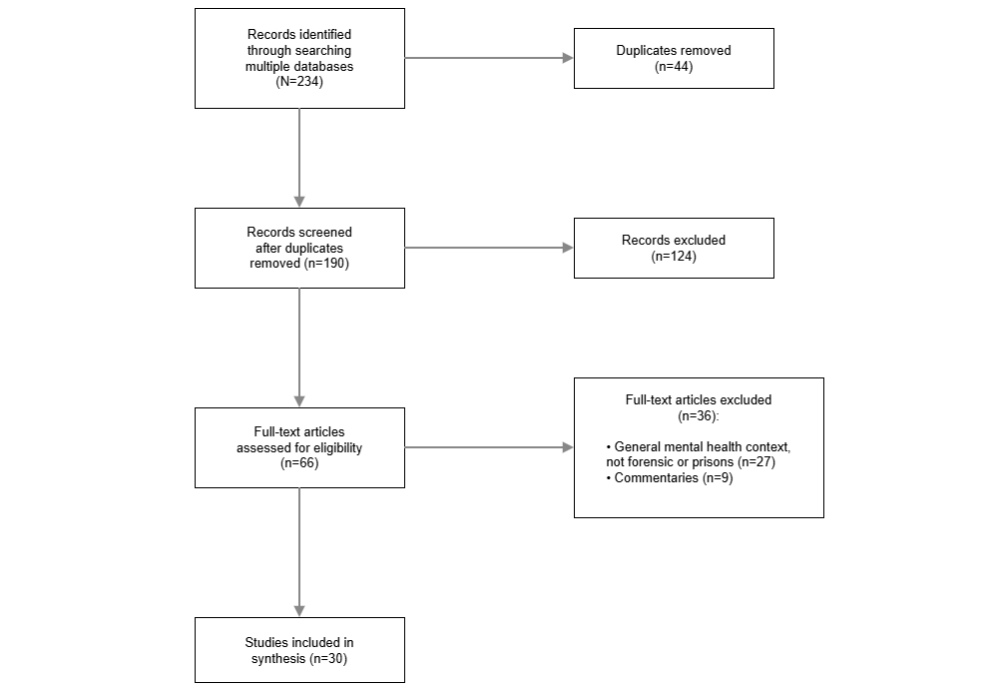
PRISMA-ScR (Preferred Reporting Items for Systematic Reviews and Meta-Analyses extension for Scoping Reviews) flow diagram.

The search results are shown in Figure 1. Table 1 provides an overview of the included sources, highlighting study design, target users, and technology type and use. It is notable that over two-thirds of the literature included were published within the previous 4 years (2022-2025), highlighting the status of immersive technology as an emerging and expanding field. A detailed overview of the characteristics of the 30 primary studies included is provided in Multimedia Appendix 1 [13,14,22-49].

Findings across the included literature were mixed. Many studies (22/30, 73.3%) reported positive outcomes, including reductions in anxiety and stress and increased relaxation [22-25]; reductions in anger, aggression, and impulsivity [14,26-30]; and improvements in well-being and empathy, as well as in skills such as leave readiness, employment readiness, and social cognition [26,31,32]. Increased engagement with training or treatment was also reported across several studies (8/30, 26.7%) [14,27,31,33-37], and 20% (6/30) described high acceptability, usability, or validity [13,29,38-41]. However, 13.3% (4/30) of the studies reported no significant differences in primary outcome measures, including aggression [42,43]; recidivism [31]; and self-efficacy, anxiety, burnout, and empathy [38].

The included studies varied considerably in design, participant group, setting, technology, and outcome measures, which limits the extent to which the findings can be compared or interpreted as evidence of effectiveness. Reporting was also inconsistent, particularly in relation to the technologies used and the terms used to describe them. In some cases, studies described web-based training agents as “VR” despite these not involving fully immersive environments [31,44]. The research frequently relied on small samples, lacked comparative controls, and rarely examined longer-term outcomes. Only 3.3% (1/30) of the studies assessed longer-term effects, reporting short-term improvements in anger control, impulsivity, and hostility but no lasting reduction in aggressive behavior [42]. The evidence base was also concentrated geographically, with most studies conducted in the Netherlands (10/30, 33.3%) and Scandinavia (8/30, 26.7%), and several focused on products developed by a single provider, CleVR BV, particularly the VR aggression prevention training (VRAPT) platform. This variability makes it difficult to draw firm conclusions about effectiveness across settings and applications.

**Table 1:** Characteristics of the included literature on immersive technology in forensic mental health services (FMHS) and prison settings (N=30).

Characteristics				Values
Publication trend				>75% published in 2022-2025
Study design, n/N (%)				
	Qualitative			13/30 (43.3)
		Co-design	3/13 (23.1)	
	Quantitative			8/30 (26.7)
		RCT^a^	5/8 (62.5)	
	Mixed methods			9/30 (30)
Primary research focus, n (%)				
	FMHS			17 (56.7)
	Prison settings			13 (43.3)
Target end users				FMHS users, incarcerated individuals, and staff
Intended use of immersive technology, n (%)				
	Therapeutic tool			14 (46.7)
	Skill training and re-entry preparation			10 (33.3)
	Clinical assessment			3 (10)
	Technology evaluation			3 (10)
Degree of immersion, n (%)				
	NIVR^b^			1 (3.3)
	SIVR^c^			3 (10)
	IVR^d^			22 (73.3)
	Views on technology use only			2 (6.7)
	Not enough details			2 (6.7)

^a^RCT: randomized controlled trial.

^b^NIVR: nonimmersive virtual reality. A desktop-based application where interaction occurs through typical 2D screens and standard input devices such as a keyboard or mouse. Users maintain a strong awareness of the physical world, resulting in minimal immersion.

^c^SIVR: semi-immersive virtual reality. A system using large projection screens and/or advanced interactive devices such as gaming controllers or haptic devices. It provides partial immersion and a heightened sense of presence while still allowing for perception of the real surroundings.

^d^IVR: immersive virtual reality. A fully enveloping virtual reality experience achieved through head-mounted displays and 3D input devices. Users perceive and interact with the virtual environment as if it were their primary reality, producing the highest level of immersion.

### Opportunities and Barriers to Implementation

Although the studies varied widely in scope, purpose, and methodological rigor, this review identified 5 broad areas of opportunity and 4 potential barriers related to how immersive tools were (or could be) taken up, used, and perceived.

#### Opportunities

The themes highlighting opportunities for immersive technology in FMHS and prison contexts were developed with the intended end users (FMHS service users and incarcerated individuals) positioned at the center of our analysis.

##### Enhanced Immersion and Engagement

Immersive technologies were widely reported to improve engagement and attention in both prison [27,33,34,37] and FMHS settings [14,35]. Several interventions adapted existing therapeutic tools into VR, including the VR spiritual emotional freedom technique for emotional regulation [25,33], VR calm rooms for relaxation [23,24], job interview training [44], and therapeutic sessions (eg, ProReal therapy interventions) [37]. These tools used features such as visual and auditory realism and detailed spatial environments to strengthen presence and attentional focus [13,22]. Across these adaptations, VR offered advantages such as heightened focus, reduced environmental distraction, and enhanced motivation through novelty and gamelike elements. In some cases, VR also enabled access to otherwise unavailable physical experiences (eg, calm rooms), thereby expanding users’ opportunities for regulated, constructive engagement. Participants across multiple studies reported enjoying the experiences brought on by the immersive technology they engaged with [14,28,31,33,45]. Users frequently emphasized the value of small environmental details (eg, window shopping in simulated streets [13]) in creating meaningful and believable experiences. Alshaer [33] highlighted the potential for VR to immerse participants beyond the controlled environments, such as visiting family or walking on a beach, offering a preparatory adaptation tool for individuals transitioning back into society.

A subset of studies (2/30, 6.7%) drew on arts-based approaches to create richer sensory and emotional engagement. Storytelling and narrative techniques were used to support empathy and expression [46], whereas the Deep VR program [28] combined immersive art with gamified diaphragmatic breathing. In Deep VR, users navigate a calming underwater world through breath-controlled movement supported by subtle visual biofeedback. Patients and staff viewed it as accessible, relaxing, and potentially simple to implement, although some noted limited long-term variety and occasionally subtle biofeedback cues. Overall, these arts-infused approaches contributed to enhancing immersion and engagement in otherwise highly controlled environments.

##### Supporting Autonomy and Agency

Several studies (8/30, 26.7%) highlighted the potential for immersive technologies, particularly VR, to enhance autonomy and agency in FMHS and prison settings. Ligthart et al [4] and Burns et al [50] reported that VR interventions can support “agential fluidity,” allowing users to make choices and act independently within the virtual environment. Studies on the Deep VR program [28] and VR calm rooms [23,24] showed that interventions could be used on a self-directed basis with minimal staff facilitation. There was also evidence suggesting that personalization such as adjusting visual features for paranoia enhances user’s sense of control and autonomy [28]. Immersion itself was reported to provide realistic, meaningful experiences that allow users to practice skills and engage therapeutically without direct supervision [13,22]. Teng and Gordon [47] demonstrated that co-design processes involving end users in consultation, workshops, and production roles further supported autonomy and reduced potential for harm. Other studies (3/30, 10%) highlighted secondary benefits such as improved dialogue with therapists [36], safe exploration of aggressive behaviors [42], and personal insight [43], suggesting that VR can promote agency even beyond its immediate therapeutic aims.

##### Co-Design and End User Involvement

Several studies (3/30, 10%) highlighted co-design as an important opportunity for developing credible, relevant, and acceptable immersive interventions. Teng and Gordon [47] demonstrated that interventions developed without end user participation risk missing the nuances of lived experience, prompting their shift toward a participatory approach. By involving formerly and currently incarcerated women in activities such as storyboarding and scripting VR scenarios, the authors were able to create content that more accurately reflected the challenges of re-entry and supported participant agency. Every aspect of the VR intervention prototype was cocreated with end users. Similarly, Hodge et al [40] showed that working directly with FMHS service users to design a serious game prototype improved ecological validity and engagement. Participants shaped realistic high-risk scenarios and gameplay pathways, and both service users and clinicians saw value in using the game for discussing high-risk situations and supporting discharge preparation. The need for participatory approaches was further underscored by van Rijn et al [37] when evaluating the ProReal VR program—a laptop-based world-building virtual environment that allows users to manipulate scenes with avatars to externalize thoughts and experiences. They revealed that, although participants found the tool useful for externalizing and communicating their thoughts, the introduction of another imposed intervention in a closed setting was met with hesitation. One participant questioned “What are they trying to put on us now?” voicing distrust about the motives behind introducing the tool. There were also concerns that personal disclosures might be shared with the prison or parole boards, highlighting the need for greater user involvement and transparency when implementing new interventions in closed, highly controlled settings.

##### Valuable Unintended Outcomes

Several studies (4/30, 13.3%) reported that immersive technologies often produced benefits beyond their intended clinical outcomes. While quantitative evaluations found limited or nonsignificant effects on primary measures such as aggression and anxiety [38,42,43], qualitative findings highlighted meaningful ancillary benefits for both service users and clinicians. For example, VR interventions were shown to facilitate dialogue between patients and therapists in FMHS settings, helping build trust where it was otherwise limited [36]. VR-assisted aggression training allowed therapists to safely explore patients’ aggressive behavior without compromising therapeutic relationships, with avatars absorbing directed aggression and preventing lasting anger toward clinicians [42]. Even when VR-based games did not reduce anger more than in control conditions, both patients and practitioners identified insights into behavioral triggers and personal reflection, such as recognizing positive intentions in others [43].

##### Personalization Supports Engagement and Safety

Personalization was identified as a consistent gap and key recommendation across studies. Authors emphasized that VR tools should be adaptable to individuals’ backgrounds, risk factors, and treatment needs, with even small customizable features viewed as clinically meaningful [27,48]. Personalization was seen as important both to enhance engagement and to promote safety for users. Multiple studies (6/30, 20%) reported that allowing users and clinicians to customize VR environments improved relevance, usability, and therapeutic impact [28,48]. For example, the DEEP VR program enabled participants to select different guided relaxation methods, fostering autonomy and independence while learning new coping mechanisms [28]. Participants in this study described relief, relaxation, and a sense of control over their experience. Personalization was also linked to enhanced perceptions of safety by allowing users moments of control in settings where decisions are often made for them while also fostering clinician confidence in the appropriateness and adaptability of VR interventions for different patient profiles [48]. Similarly, Ilioudi et al [23,24] suggested that VR calm rooms be adapted with more personalized content, such as social or animal-based environments, to better meet user needs. Trahan et al [51] argued that co-ownership of VR programs allows for more adaptable and flexible applications and iterative adjustment based on the user as issues arise without the constraints typically associated with commercially owned software.

#### Barriers

Four themes associated with barriers to the implementation of immersive technologies in FMHS and prison settings were identified. These were structural and institutional factors, ethical and human rights concerns, limited end user involvement, and technical and personalization limitations.

##### Structural and Institutional Constraints

Multiple structural and institutional challenges limit the implementation of immersive technologies in FMHS and prison settings. Access to participants and research sites is often restricted, making it difficult to conduct studies with sufficient sample sizes or rigorous quantitative designs [42,47]. In addition, institutional priorities and rules can conflict with research and the introduction of new tools, creating tensions between best research practice and existing strict rules and social controls [47]. Kouijzer et al [13] noted that successful VR implementation in clinical settings relies not just on technology access but also on organizational support and structured staff training. Health care providers in their study emphasized the importance of introductory materials, peer learning, and hands-on practice to build confidence [13]. Staff training using VR has been shown to significantly reduce restrictive practices compared to usual treatment [38]. Providing dedicated time, guidance, and management endorsement is therefore critical, particularly given limited evidence on safety and efficacy for certain populations, making careful, staff-focused implementation a necessary first step.

##### Ethical and Human Rights Concerns

Ethical and human rights considerations were identified as a significant theme across the reviewed literature, with immersive technologies often described as operating in an ethical “grey area” in FMHS and prison settings [4]. Ethical scholars caution that immersive technologies should not be seen as “moral prostheses” that automatically improve behavior or judgment [4]. Rather, they are “experience machines” that can influence behavior in intended and unintended ways [52]. In contexts with vulnerable users and power imbalances, such tools should be introduced carefully, with strong protection, clear evidence, and strict limits to avoid coercion or misuse [53]. Greenbaum [54] further highlights the human rights risks of VR in prisons, including pervasive monitoring, collection of sensitive biometric and behavioral data, potential manipulation of memories, and exposure to psychologically distressing or punitive experiences. Concerns about consent, confidentiality, coercion, dependency, and the risk that immersive tools could reinforce institutional control or negatively affect mental states all underline the delicate ethical balance required when introducing immersive tools into these settings [50,54-56]. Across the literature, these tensions point to the need for cautious, well-governed implementation and greater empirical evidence to ensure that immersive technologies uphold rather than compromise the rights and well-being of those in forensic and custodial environments.

##### Limited or Late End User Involvement

A further barrier identified was the limited involvement of end users in intervention design. Several studies (4/30, 13.3%) did not engage participants at the earliest stages of design, instead seeking feedback on preexisting VR applications or interventions [4,47]. Not all service users had a positive attitude toward technology in the studies we identified. When consulted, participants often reported fatigue or frustration from repeated involvement in research projects, indicating that end user perspectives were frequently treated as an afterthought [37,47]. Compensation for participation was rarely provided, with only 3.3% (1/30) of the studies reporting the use of small vouchers redeemable within the facility [36]. Late end user involvement limits the extent to which service users can shape the assumptions, scenarios, interaction features, and safeguards embedded in immersive interventions. This may produce tools that insufficiently reflect users’ lived experiences, institutional constraints, trauma histories, privacy concerns, or support needs, thereby reducing their perceived relevance, acceptability, safety, and practical usefulness.

##### Technical and Personalization Limitations

Several studies (9/30, 30%) highlighted the lack of personalization or customization in VR interventions, reducing their relevance to individual patient needs [12,28,41,49]. In one study, while the VR environment was perceived as realistic and engaging and the VRAPT was perceived as effective by some incarcerated individuals, others believed that the VR experience lacked realism and did not work for them [45]. Both health care providers and service users expressed a wish for enhanced realism and reduced physical discomfort while immersed in VR [13]. Cybersickness was reported as a concern in one study [14] but considered minimal in another [26]. Environmental details in virtual spaces were sometimes insufficient to create realistic, lifelike experiences, which may reduce therapeutic impact [13]. Furthermore, the use of commercially available or rigid VR platforms limited adaptability, constraining the ability of clinicians to tailor experiences for different clinical presentations or treatment plans [51]. These technical constraints highlight the importance of flexible, adaptable, and user-informed design in immersive technology applications.

## Discussion

### Principal Findings

This scoping review identified 30 primary studies examining the use of immersive technologies in FMHS and prison settings. The field remains highly exploratory, characterized by considerable variation in aims, methods, and reported outcomes.

Most interventions (24/30, 80%) relied on VR, with few tailored to specific clinical or prison populations with limited guidance for selecting appropriate technologies, content, or outcome measures. The evidence base remains too fragmented to draw firm conclusions regarding clinical benefit, safety, or feasibility, particularly over the long term.

The evidence base identified in this review was highly heterogeneous in terms of setting, participant group, technology type, study design, and outcome measures. Studies included work in both forensic mental health and prison settings; used a range of immersive technologies with different levels of immersion; and examined varied purposes, including treatment, skill training, assessment, and attitudes toward technology use. This breadth is useful for mapping an emerging area, but it also limits the strength of the conclusions that can be drawn. The themes identified in this review highlight patterns across the literature rather than evidence of consistent effectiveness, and findings should be interpreted with the variation in source types and evidential weight in mind.

The evidence base was also concentrated in a relatively small number of geographical settings, with much of the literature coming from Dutch and Scandinavian contexts. In addition, several studies (10/30, 33.3%) drew on applications developed by a single provider, CleVR BV, which further narrows the range of technologies and intervention models represented in the literature. This concentration limits the generalizability of the findings and makes it difficult to know how well the reported benefits, barriers, and implementation issues would transfer to other jurisdictions, services, or digital platforms.

In relation to RQ 1, the reviewed literature focused mainly on therapeutic uses of immersive technologies, particularly in relation to aggression, anxiety, stress, relaxation, and emotion regulation, alongside a smaller body of work on skill training in areas such as leave readiness, employment, literacy, vocational learning, and social cognition. A more limited number of studies (3/30, 10%) examined assessment uses, and only 3.3% (1/30) of the studies focused on staff training. FMHS-based studies tended to focus on clinical and therapeutic outcomes, whereas prison-based studies more frequently examined educational, vocational, and re-entry applications, reflecting the differing frameworks of treatment and rehabilitation, respectively. This suggests that the current research landscape is weighted toward individual-level treatment and skill-based applications, with less attention paid to implementation in routine care, staff development, service-level outcomes, and longer-term follow-up.

Despite this, immersive technologies are being trialed for a wide range of purposes, including therapeutic interventions, skill training, educational activities, and ways to connect service users and incarcerated individuals with environments beyond their immediate settings. Interest from both service users and staff was evident, particularly where tools were codeveloped with end users. However, environmental restrictions, limited access to equipment, and institutional barriers frequently constrained participation and prevented rigorous evaluation. The dominance of a single provider (CleVR BV) and concentration of research in one geographical region contributed to a narrow methodological base, limiting insights into how different design features, interaction styles, or levels of immersion influence engagement or outcomes. These patterns also underscore the need for increased clarity in terminology and improved digital literacy among clinicians, researchers, and decision-makers.

The theme of valuable unintended outcomes also speaks directly to RQ 2 and was one of the more interesting findings of the review. Across several studies (4/30, 13.3%), immersive technologies appeared to offer benefits that were not always captured by the main outcome measures used, including improved engagement and greater self-awareness, empathy, insight, and willingness to take part in treatment or training. This suggests that the value of immersive technologies in forensic mental health and prison settings may not lie only in symptom reduction or measurable behavior change but also in their potential to support reflection, involvement, and readiness to engage. At the same time, these findings were often reported as secondary or qualitative observations rather than as primary outcomes and should therefore be interpreted with caution. Further research is needed to examine whether these effects can be measured more consistently and whether they lead to meaningful change over time.

### Limitations

Several limitations should be considered when interpreting the findings of this review. First, no review protocol was prospectively registered. Although this reflected the exploratory purpose of the scoping review and the need to map an emerging and heterogeneous evidence base, the absence of a registered protocol limits transparency regarding any methodological decisions made during the review process.

Second, interrater reliability statistics were not calculated for study selection. Screening decisions were managed through predefined eligibility criteria, Covidence, and consensus discussion among the review team. However, the absence of formal agreement statistics limits the extent to which reviewer consistency can be independently assessed.

Third, this review was limited to English-language publications indexed in Scopus, PubMed, and CINAHL supplemented by reference list searching. Relevant studies published in other languages, indexed in other databases, or available only through unpublished or local institutional reports may therefore have been missed.

Finally, the evidence base was geographically and technologically concentrated. Many studies (17/30, 56.7%) were conducted in Dutch and Scandinavian contexts, whose forensic mental health, prison, rehabilitation, procurement, and governance structures may not be directly transferable to other jurisdictions. In addition, several studies (10/30, 33.3%) examined technologies developed by or associated with a small number of providers, particularly CleVR BV and VRAPT-related applications. This narrows the range of platforms, design approaches, interaction styles, and implementation models represented in the literature. Although conflicts of interest were not formally assessed, this concentration highlights the importance of transparent reporting of technology ownership, developer involvement, funding relationships, and researcher independence in future research.

### Contribution to the Literature

While aligned with previous reviews, our work extends the literature in several important ways. First, it specifically focused on high-security, legally complex environments where the constraints and ethical considerations differ markedly from those of wider health care, general mental health, or educational contexts. Second, unlike previous reviews that centered primarily on VR, our synthesis incorporated a broader range of immersive technologies and a more diverse set of use cases. Third, by examining the intersection of mental health, prison environments, and emerging technology, we highlighted additional structural barriers, including strict environmental controls, institutional policies, and safety protocols, that shape implementation in ways not fully addressed by earlier work.

Finally, and most importantly, our findings emphasize the need for coproduction with service users and staff; greater technological diversity; and clearer guidelines for tool selection, implementation, and evaluation. Immersive technologies for staff training remain relatively untapped yet hold strong potential for future exploration. Addressing these gaps will be essential to introduce these tools ethically and safely while supporting rehabilitation, autonomy, and well-being in FMHS and prison contexts.

Our findings closely align with the conclusions of prior literature reviews across related domains. Consistent with the work by Kip et al [18], we found that immersive interventions must be integrated into existing FMHS pathways rather than added as isolated components. Seamless integration, ongoing evaluation, and structured implementation support remain critical needs. Similar to the review by Cushnan et al [12], our findings reinforce that, although immersive technologies hold potential across a broad spectrum of psychological and cognitive challenges, their adoption is hindered by the gap between technological innovation and clinical readiness. Clinicians’ perceptions, familiarity, and confidence with these tools continue to shape uptake.

The potential identified in prison settings mirrors the conclusions by Pires et al [17], where VR was recognized as promising yet still in its early developmental stage, offering opportunities for rehabilitation, safe skill practice, and enhancing risk assessment and reintegration efforts. Our findings also echo the observations by Sygel and Wallinius [16]: the predominance of small-scale, nonrandomized studies; limited personalization of simulations; broad methodological variability; and the near absence of long-term follow-up. As in their review, we found no evidence of major adverse effects but also insufficient evidence to support wide-scale implementation of any specific immersive intervention at present.

### Conclusions

This review highlights opportunities for immersive technologies in FMHS and prison settings, but the evidence remains too limited for wide-scale adoption. New tools risk being introduced as “quick fixes” without careful consideration of implementation or unintended consequences. Strengthening the evidence base, planning implementation carefully, and involving end users throughout will be essential to ensure ethical, safe, and beneficial use. The development of formal implementation protocols and minimum safety standards represents a clear priority for future empirical and clinical work.

## Appendix

Multimedia Appendix 1 Descriptive overview of the 30 primary studies included in the scoping review, detailing study characteristics, immersive technologies used, and key findings.

Multimedia Appendix 2 PRISMA-ScR checklist.

## Abbreviations

FMHS: forensic mental health services
JBI: Joanna Briggs Institute
RQ: research question
VR: virtual reality
VRAPT: virtual reality aggression prevention training

## References

[1] Madanian S, Nakarada-Kordic I, Reay S, Chetty T (2023). Patients' perspectives on digital health tools. PEC Innov.

[2] Colecchia F, Giunchi D, Qin R, Ceccaldi E, Wang F (2025). Editorial: machine learning and immersive technologies for user-centered digital healthcare innovation. Front Big Data.

[3] Slater M (2009). Place illusion and plausibility can lead to realistic behaviour in immersive virtual environments. Philos Trans R Soc Lond B Biol Sci.

[4] Ligthart S, Meynen G, Biller-Andorno N, Kooijmans T, Kellmeyer P (2022). Is virtually everything possible? The relevance of ethics and human rights for introducing extended reality in forensic psychiatry. AJOB Neurosci.

[5] Olawade DB, Ayoola FI, Ebo TO, Asaolu AJ, Egbon E, Clement David-Olawade A (2025). Artificial intelligence in forensic mental health: a review of applications and implications. J Forensic Leg Med.

[6] McKenna B, Maguire T, Martin T, Evans K, Nizette D, O'Brien A (2016). Forensic mental health nursing. Psychiatric and Mental Health Nursing.

[7] Martin T, Ryan J, Bawden L, Maguire T, Quinn C, Summers M (2012). Forensic mental health nursing: standards of practice 2012. Victorian Institute of Forensic Mental Health (Forensicare).

[8] Lutz M, Zani D, Fritz M, Dudeck M, Franke I (2022). A review and comparative analysis of the risk-needs-responsivity, good lives, and recovery models in forensic psychiatric treatment. Front Psychiatry.

[9] Tully J, Hafferty J, Whiting D, Dean K, Fazel S (2024). Forensic mental health: envisioning a more empirical future. Lancet Psychiatry.

[10] Freeman M (2022). The World Mental Health Report: transforming mental health for all. World Psychiatry.

[11] Minerva F, Giubilini A (2023). Is AI the future of mental healthcare?. Topoi (Dordr).

[12] Cushnan J, McCafferty P, Best P (2024). Clinicians' perspectives of immersive tools in clinical mental health settings: a systematic scoping review. BMC Health Serv Res.

[13] Kouijzer MT, Kip H, Kelders SM, Bouman YH (2024). The introduction of virtual reality in forensic mental healthcare - an interview study on the first impressions of patients and healthcare providers regarding VR in treatment. Front Psychol.

[14] González Moraga FR, Enebrink P, Perrin S, Sygel K, Veling W, Wallinius M (2024). VR-assisted aggression treatment in forensic psychiatry: a qualitative study in patients with severe mental disorders. Front Psychiatry.

[15] Kouijzer MM, Kip H, Bouman YH, Kelders SM (2023). Implementation of virtual reality in healthcare: a scoping review on the implementation process of virtual reality in various healthcare settings. Implement Sci Commun.

[16] Sygel K, Wallinius M (2021). Immersive virtual reality simulation in forensic psychiatry and adjacent clinical fields: a review of current assessment and treatment methods for practitioners. Front Psychiatry.

[17] Pires AR, Fernandes Â, Estalella G, Zisiadou M, Carrolaggi P, Loja S, Leitão T (2021). The potential of virtual reality for education and training in prisons. Virtual Reality to Train Inmates.

[18] Kip H, Bouman YH, Kelders SM, van Gemert-Pijnen LJ (2018). eHealth in treatment of offenders in forensic mental health: a review of the current state. Front Psychiatry.

[19] Mak S, Thomas A (2022). Steps for conducting a scoping review. J Grad Med Educ.

[20] Aromataris E, Lockwood C, Porritt K, Pilla B, Jordan Z (2024). JBI Manual for Evidence Synthesis.

[21] Mays N, Roberts E, Popay J, Allen P, Black N, Clarke A, Fulop N, Anderson S (2001). Synthesising research evidence. Studying the Organisation and Delivery of Health Services: Research Methods.

[22] Bakkarang S, Saleh A, Syamsuddin S, Erika KA, Nursalam N, Bukhari A, Arafat R, Lisal ST, Firmansyah F (2023). The effect of virtual reality spiritual emotional freedom technique (Vr-seft) therapy on anxiety and cortisol in drug patients in Makassar Class I State Detention Center. Multidiscip Sci J.

[23] Ilioudi M, Lindner P, Ali L, Wallström S, Thunström AO, Ioannou M, Anving N, Johansson V, Hamilton W, Falk Ö, Steingrimsson S (2023). Physical versus virtual reality-based calm rooms for psychiatric inpatients: quasi-randomized trial. J Med Internet Res.

[24] Ilioudi M, Wallström S, Steingrimsson S, Lindner P, Thunström AO, Ali L (2023). Patient experience of a virtual reality calm room in a psychiatric inpatient care setting in Sweden: a qualitative study with inpatients. BMJ Open.

[25] Saleh A, Syamsuddin S, Erika KA, Bukhari A, Arafat R, Lisa ST, Tahe R, Thalib A, Saharullah, Nursalam, Firmansyah (2024). Virtual reality-based Spiritual Emotional Freedom Technique (SEFT) model on spiritual well-being and BDNF levels in drug inmates in the class 1 state prison in Makassar. Pak J Life Soc Sci.

[26] Hendriks C, Jansen JM, Smit M, Smulders LM, Popma A, Van Der Pol T (2023). VReedom: training for authorized leave of absence through virtual reality - a feasibility study. Front Psychol.

[27] Ivarsson D, Delfin C, Enebrink P, Wallinius M (2023). Pinpointing change in virtual reality assisted treatment for violent offenders: a pilot study of Virtual Reality Aggression Prevention Training (VRAPT). Front Psychiatry.

[28] Klein Haneveld L, Kip H, Bouman YH, Weerdmeester J, Scholten H, Kelders SM (2023). Exploring the added value of virtual reality biofeedback game DEEP in forensic psychiatric inpatient care-a qualitative study. Front Psychol.

[29] Sappelli F, Lobbestael J, van Haalen DL, Böckmann I, Bulten BE, Verkes RJ (2025). Virtual reality aggression assessment with social interaction: early evidence for validity from two pilot studies. Front Psychol.

[30] Woicik K, Geraets CN, Klein Tuente S, Masthoff E, Veling W (2023). Virtual reality aggression prevention treatment in a Dutch prison-based population: a pilot study. Front Psychol.

[31] Smith MJ, Parham B, Mitchell J, Blajeski S, Harrington M, Ross B, Johnson J, Brydon DM, Johnson JE, Cuddeback GS, Smith JD, Bell MD, McGeorge R, Kaminski K, Suganuma A, Kubiak S (2023). Virtual reality job interview training for adults receiving prison-based employment services: a randomized controlled feasibility and initial effectiveness trial. Crim Justice Behav.

[32] Sivermo F, Moraga FR, Wallinius M (2025). A pilot study on treatment content in virtual reality-assisted aggression therapy at a maximum-security forensic psychiatric clinic. Sci Rep.

[33] Alshaer A (2023). Virtual reality in training: a case study on investigating immersive training for prisoners. Int J Adv Comput Sci Appl.

[34] Collins J, Langlotz T, Regenbrecht H (2020). Virtual reality in education: a case study on exploring immersive learning for prisoners. Proceedings of the 2020 IEEE International Symposium on Mixed and Augmented Reality Adjunct.

[35] Dumont M, Briand C, Aubin G, Dumais A, Potvin S (2022). Developing immersive videos to train social cognition in individuals with schizophrenia in forensic psychiatry. J Forensic Pract.

[36] Hedström R, Wallinius M, Sygel K, Geraets CN (2023). Virtual reality-assisted assessment of paranoid ideation in forensic psychiatric inpatients: a mixed-methods pilot study. Front Psychol.

[37] van Rijn B, Cooper M, Jackson A, Wild C (2015). Avatar-based therapy within prison settings: pilot evaluation. Br J Guid Couns.

[38] Phiri P, Pemberton L, Liu Y, Yang X, Salmon J, Boulter I, Sajid S, Clarke J, McMillan A, Shi JQ, Delanerolle G (2024). Tree: reducing the use of restrictive practices on psychiatric wards through virtual reality immersive technology training. World J Psychiatry.

[39] Claborn KR, Conway F, Nydegger LA (2024). Acceptability and perceived utility of virtual reality among people who are incarcerated who use drugs. J Correct Health Care.

[40] Hodge P, Davis J, Maiden N, Mann B, Nidsjo A, Simpson A, Reynolds L (2015). StreetWise: a valid ecology for a serious game in a secure forensic mental health setting. Procedia Comput Sci.

[41] Kip H, Kelders SM, Weerink K, Kuiper A, Brüninghoff I, Bouman YH, Dijkslag D, van Gemert-Pijnen LJ (2019). Identifying the added value of virtual reality for treatment in forensic mental health: a scenario-based, qualitative approach. Front Psychol.

[42] Klein Tuente S, Bogaerts S, Bulten E, Keulen-de Vos M, Vos M, Bokern H, IJzendoorn SV, Geraets CN, Veling W (2020). Virtual Reality Aggression Prevention Therapy (VRAPT) versus waiting list control for forensic psychiatric inpatients: a multicenter randomized controlled trial. J Clin Med.

[43] Smeijers D, Bulten EH, Verkes RJ, Koole SL (2021). Testing the effects of a virtual reality game for aggressive impulse management: a preliminary randomized controlled trial among forensic psychiatric outpatients. Brain Sci.

[44] Smith MJ, Harrington M, Ross B, Quinn CR, Musan LP, Brydon DM, Johnson JE, Cuddeback GS, Smith JD, Merle JL, Burke-Miller JK, Jordan N, Bell MD, Friedman B, Kryscio P, Suganuma A (2025). A pragmatic randomized controlled trial of virtual reality job interview training in prison employment services. J Exp Criminol.

[45] Ivarsson D, Enebrink P, Delfin C, Wallinius M (2025). Different worlds: a qualitative study on the use of VR treatment in prison to change for the real world. Vict Offenders.

[46] Oikonomou K, Gerostergiou K, Liovas D, Stathopoulos C, Liakos E, Kolokotronis D, Malamos AG, Lisitsa E, Trantas G, Papadakis K, Anagnostopoulou K (2020). Virtual reality in humanistic prisons education. The STEPS project. Proceedings of the 24th Pan-Hellenic Conference on Informatics.

[47] Teng MQ, Gordon E (2021). Therapeutic virtual reality in prison: participatory design with incarcerated women. New Media Soc.

[48] Kip H, Oberschmidt K, Bierbooms JJ (2021). eHealth technology in forensic mental healthcare: recommendations for achieving benefits and overcoming barriers. Int J Forensic Ment Health.

[49] Wit-de Visser B, Rijckmans MJ, Vermunt JK, Hamakers MJ, van Dam A (2026). Profiles of mentalizing in individuals with antisocial behavior: comparing state- and trait-mentalizing. Psychother Res.

[50] Burns MB, Lebkuecher G, Rahman S, Roytman M, Samoska S, Vukov J (2022). Extended frameworks for extended reality: ethical considerations. AJOB Neurosci.

[51] Trahan MH, Smith KS, Traylor AC, Washburn M, Moore N, Mancillas A (2019). Three-dimensional virtual reality: applications to the 12 grand challenges of social work. J Technol Hum Serv.

[52] Rueda J, Dore-Horgan E (2022). A virtual prosthesis for morality? Experiential learning through XR technologies for autonomy enhancement of psychiatric offenders. AJOB Neurosci.

[53] Moerenhout T (2022). We're not on a holodeck, yet. A social experiment approach to introducing extended reality in forensic psychiatry. AJOB Neurosci.

[54] Greenbaum D (2022). VR in the prison system: ethical and legal concerns. AJOB Neurosci.

[55] Blitz MJ (2022). Extended reality, mental liberty, and state power in forensic settings. AJOB Neurosci.

[56] Fyfe S, Lanphier E, Peterson A (2022). Neurorights for incarcerated persons: should we curb inflation?. AJOB Neurosci.

